# MicroRNA Signatures in Cardiovascular Diseases: A Systematic Literature Review

**DOI:** 10.7759/cureus.110938

**Published:** 2026-06-15

**Authors:** Hariballav Mahapatra, Pankaj Banotra, Anand Sekar G, Jignesh Sharma, Sharat Vishwanath K, Dinesh Tripathi

**Affiliations:** 1 Department of Diabetology, Sevayan Diabetes Centre, Puri, IND; 2 Department of Cardiology, Sri Jayadeva Institute of Cardiovascular Sciences, Bangalore, IND; 3 Department of Cardiology, Aarupadai Veedu Medical College, Vinayaka Missions Research Foundation, Kirumampakkam, IND; 4 Department of Pediatrics, Mansarovar Medical College, Sehore, IND; 5 Department of Mental Health Nursing, Bhopal Memorial Hospital and Research Centre, Bhopal Nursing College, Bhopal, IND; 6 Department of Biochemistry, Subharti Medical College, Meerut, IND

**Keywords:** acute myocardial infarction, biomarkers, cardiovascular disease, coronary artery disease, microrna

## Abstract

Cardiovascular diseases remain the leading contributors to global morbidity and mortality, and current diagnostic and prognostic tools do not fully capture disease-specific molecular changes. MicroRNAs (miRNAs) are small non-coding RNAs that regulate gene expression and are increasingly being investigated as stable circulating biomarkers in cardiovascular disorders. This systematic review aimed to qualitatively synthesise evidence on microRNA signatures associated with cardiovascular diseases and evaluate their diagnostic, prognostic, predictive, severity-related, and treatment-response relevance. A systematic search was conducted in PubMed/MEDLINE, Scopus, Web of Science, ScienceDirect, and Google Scholar using microRNA-, cardiovascular disease-, and biomarker-related terms. Original studies reporting extractable microRNA findings in cardiovascular disease populations or clinically relevant translational models with human validation were included. Review articles were used only for background context. Eleven eligible studies were qualitatively synthesised; no meta-analysis was performed because of heterogeneity in cardiovascular phenotypes, biospecimens, analytical platforms, and outcomes. The review found that plasma and serum microRNA signatures were associated with acute myocardial infarction, coronary artery disease, postoperative atrial fibrillation, heart failure, HFpEF, pulmonary arterial hypertension, coronary artery calcification, and cardiovascular risk burden. Multi-microRNA panels generally showed stronger clinical performance than single markers. The evidence base was limited by heterogeneity in study populations, cardiovascular phenotypes, biospecimen sources, miRNA extraction and profiling platforms, normalisation approaches, outcome definitions, and limited standardisation across miRNA platforms. Overall, microRNA signatures represent promising minimally invasive biomarkers for cardiovascular disease characterisation, although standardised validation remains essential before clinical implementation.

## Introduction and background

Cardiovascular diseases remain an important cause of morbidity and mortality worldwide, and their clinical spectrum includes subclinical accumulation of risk factors, acute myocardial infarction (MI), coronary artery disease (CAD), heart failure (HF), atrial fibrillation (AF), pulmonary arterial hypertension (PAH), and vascular calcification (VC) [1]. Although conventional diagnostic and prognostic tools, including imaging, biochemical markers, clinical scoring systems, and haemodynamic assessment, have improved cardiovascular risk evaluation, they do not fully capture disease-specific molecular activity [2]. Many cardiovascular disorders are still characterised by diagnostic uncertainty, delayed recognition, unpredictable clinical progression, and limited molecular stratification [3]. These limitations have increased interest in molecular biomarkers that reflect biological processes underlying cardiovascular injury, including endothelial dysfunction, vascular inflammation, oxidative stress, myocardial fibrosis, adverse remodelling, cardiometabolic dysregulation, and response to therapy [4].

MicroRNAs (miRNAs) are small non-coding RNA molecules that regulate gene expression post-transcriptionally by binding to target messenger RNAs and influencing transcript degradation or translational repression [5]. In cardiovascular biology, miRNAs act as upstream regulators of interconnected pathogenic pathways rather than passive disease markers. Dysregulated miRNA expression can alter endothelial nitric oxide signalling, vascular smooth muscle cell proliferation and migration, inflammatory cytokine activity, oxidative stress responses, lipid metabolism, cardiomyocyte survival, angiogenesis, myocardial remodelling, and fibrotic signalling [3]. These mechanisms are directly relevant to atherosclerosis, plaque instability, ischaemic myocardial injury, heart failure progression, arrhythmogenic substrate formation, pulmonary vascular remodelling, and cardiometabolic disease burden. The ability to detect circulating miRNAs using molecular platforms such as quantitative PCR, microarray profiling, and next-generation sequencing, together with their stability in plasma and serum and resistance to degradation, makes them particularly relevant for biomarker development [6,7]. Studies in cardiovascular cohorts have demonstrated altered miRNA expression across multiple disease phenotypes, supporting their potential role in diagnosis, prognosis, disease classification, risk stratification, and treatment monitoring [8].

Compared with other emerging molecular biomarkers, such as proteomic and epigenetic markers, miRNAs offer distinct but complementary advantages. Proteomic biomarkers may provide more direct information about effector molecules and downstream disease activity, while epigenetic markers may reflect longer-term regulatory changes influencing gene expression [9]. By contrast, circulating miRNAs can capture upstream post-transcriptional regulation, are relatively stable in plasma and serum, and may integrate signals from endothelial dysfunction, inflammation, oxidative stress, fibrosis, angiogenesis, and cardiometabolic stress [5]. These features make miRNAs attractive for minimally invasive cardiovascular biomarker research [10]. However, their clinical implementation remains limited by several translational challenges, including heterogeneity in biospecimen processing, miRNA extraction, assay platforms, normalisation methods, disease definitions, and validation strategies [6,10]. In addition, altered miRNA expression does not always directly correspond to protein-level changes or clinical symptom expression, which means that miRNA signatures should be interpreted alongside proteomic, epigenetic, imaging, and conventional clinical biomarkers rather than as standalone diagnostic tools.

Recent reports have identified miRNA signatures associated with specific cardiovascular diseases and clinical outcomes [9]. Plasma extracellular miRNAs have been connected with cardiovascular risk factors and mortality in large population-based cohort studies, suggesting that they are relevant not just for the diagnosis of a single disease [10]. Acute MI has been associated with circulating miRNA profiles with high diagnostic value, as well as with cardiac troponin levels and survival outcomes. miRNA panels have been reported in studies of CAD that can differentiate early-onset and late-onset disease, and miRNAs have been associated with angiographic severity and metabolic risk [11]. Studies on postoperative AF have indicated the potential of circulating and atrial tissue miRNAs as indicators of arrhythmia development after coronary artery bypass grafting (CABG) [12]. HFpEF (heart failure with preserved ejection fraction) and HF (heart failure) have also been linked to miRNA deregulation, endothelial dysfunction, ventricular dysfunction, and response to treatment [13]. PAH research has provided new diagnostic and mechanistic clues by connecting circulating miRNA profiles to vascular endothelial dysfunction and BMPR2-associated pathways [14].

Despite this growing body of evidence, there are still areas of limitation to clinical translation. There are significant variations in the size of existing cohort studies, cardiovascular phenotype, type of biospecimen, miRNA extraction method, normalisation strategy, analytical platform, and definition of outcomes. A few studies have reported findings from widespread sequencing-based discovery, while others have evaluated some targeted miRNAs through targeted assays. There are a few studies that employ small exploratory sample sizes, which may pose concerns about overfitting, external validity, and replicability. While area-under-the-curve values are common foci of diagnostic studies, direct comparisons are challenging because of differences in control groups, disease stage, disease burden and comorbidity, and differences in validation design. Compared with diagnostic evidence, the evidence for prognosis and/or treatment response is less mature, and there are fewer studies incorporating miRNA signatures with functional pathway analysis or independent validation.

A systematic synthesis is required to define the ways miRNA signatures have been explored and investigated in different cardiovascular diseases, define the most common end-points, and define the areas where evidence is still lacking. In this context, a qualitative synthesis is especially appropriate in view of the heterogeneity of cardiovascular conditions, specimen sources, laboratory platforms, and statistical reporting. This synthesis can reveal common themes, identify target biomarkers for further study, detect methodological weaknesses, and assist in future validation strategies for the development of miRNAs as cardiovascular biomarkers.

Objectives of the review

This review aims to synthesise evidence on miRNA signatures reported across cardiovascular diseases and evaluate their diagnostic, prognostic, predictive, severity-related, and treatment-response relevance. The review also aims to summarise study characteristics, key findings, methodological quality, and risk-of-bias patterns across the included studies.

## Review

Methodology

Study Design

This systematic literature review aimed to synthesise evidence on miRNA signatures in cardiovascular diseases. The review was limited to original studies that assessed circulating, blood-based, plasma, serum, or tissue-associated miRNAs as diagnostic, prognostic, predictive, severity-related, risk-stratification, and treatment-response markers. Qualitative synthesis was performed for the included studies due to the different cardiovascular conditions, population characteristics, biological samples, miRNA detection platforms, and outcome measures reported in the studies. This clinical and methodological heterogeneity precluded the possibility of a meta-analysis. Review articles were not included in the main findings, but relevant review articles were used to support the introduction and discussion.

Search Strategy

A structured electronic database search was conducted to identify relevant studies on miRNA signatures in cardiovascular diseases. The final literature search was conducted on April 30, 2026. Searches covered records available from database inception to April 30, 2026. The main databases searched were PubMed/MEDLINE, Scopus, Web of Science, ScienceDirect, and Google Scholar. Search terms combined miRNA-related, cardiovascular disease-related, and biomarker-related concepts using Boolean operators. The representative search string was: (“microRNA” OR “miRNA” OR “circulating microRNA” OR “microRNA signature” OR “miRNA signature”) AND (“cardiovascular disease” OR “coronary artery disease” OR “acute myocardial infarction” OR “myocardial infarction” OR “heart failure” OR “HFpEF” OR “postoperative atrial fibrillation” OR “atrial fibrillation” OR “pulmonary arterial hypertension” OR “coronary artery calcification” OR “vascular calcification”) AND (“biomarker” OR “diagnostic biomarker” OR “prognostic biomarker” OR “predictive biomarker” OR “risk stratification” OR “disease severity” OR “treatment response”). Search strings were adapted according to the syntax requirements of each database. Google Scholar was searched using simplified combinations of the same core terms, and the first relevant records were screened according to title and abstract relevance. Reference lists of relevant reviews and eligible full-text studies were also screened to identify additional potentially relevant articles. The study selection process followed the PRISMA (Preferred Reporting Items for Systematic Reviews and Meta-Analyses) framework shown in the attached flow diagram. Eleven eligible original studies were included.

Study Selection Process

The selection of studies was conducted in accordance with the PRISMA procedure, including identification, screening, eligibility assessment, and inclusion. After duplicate removal, titles and abstracts were screened independently by two reviewers against predefined eligibility criteria. Records considered potentially eligible by either reviewer were advanced to full-text review to minimise inappropriate exclusion during screening. Full-text articles were then assessed independently by two reviewers according to the inclusion and exclusion criteria. Disagreements during title/abstract screening or full-text eligibility assessment were resolved through discussion and consensus, with third-reviewer input when required. Studies were included only if they directly assessed miRNA expression or miRNA signatures in relation to cardiovascular disease outcomes. Articles were excluded if they lacked original data, did not report miRNA-specific findings, lacked a clearly defined cardiovascular disease context, or were not relevant to cardiovascular biomarker assessment. Studies published in languages other than English were also excluded. Studies included in the final synthesis were subjected to qualitative narrative analysis and table-based comparison.

Inclusion Criteria

Studies were selected as long as they reported original research findings on miRNA expression or miRNA signatures in cardiovascular disease populations or clinically relevant translational models with human validation. Cardiovascular conditions eligible for inclusion in the study were acute MI, CAD, postoperative AF, HF, HFpEF, PAH, cardiovascular risk-factor burden, and coronary artery calcification. Studies had to report at least one of the following extractable biomarker-related outcomes: diagnosis, prognosis, prediction, disease severity, risk stratification, or treatment response. The other criteria for eligible studies included population description, biological sample, miRNA detection method, and the main findings.

Exclusion Criteria

Monographic studies were excluded if they were reviews, meta-analyses, editorials, commentaries, letters lacking original data, or conference abstracts lacking sufficient extracted data. Review articles were not included in the main results, but relevant reviews were used in the background discussion. Studies that involved only animals were not included unless they had clinical validation that was relevant to cardiovascular disease. Studies that lacked miRNA-specific outcomes, did not adequately describe the sample source or method of analysis, or did not have a clear definition of cardiovascular disease were also excluded.

Data Extraction and Analysis

Data were extracted using a structured extraction form designed for this review. Extracted variables included author and year, study design, study population, cardiovascular condition, biological specimen, miRNA detection method, identified miRNA signature, validation or replication status, and key clinical outcome. Diagnostic, prognostic, predictive, severity-related, risk-stratification, and treatment-response findings were recorded when applicable. Data extraction was conducted independently by two reviewers, and discrepancies were resolved by consensus, with third-reviewer input when required. Narrative analysis was chosen because pooling was not suitable due to heterogeneity in study design, cardiovascular phenotype, biological sample, assay platform, validation strategy, and outcome reporting. Findings were organised by cardiovascular condition and clinical application to support the preparation of the integrated study characteristics and key findings table.

Quality and Risk of Bias Assessment

The quality of the studies and risk of bias were evaluated using an adapted domain-based framework suitable for biomarker-focused observational and diagnostic studies. Assessment principles were drawn from QUADAS-2 (Quality Assessment of Diagnostic Accuracy Studies-2) and the Newcastle-Ottawa Scale (NOS) [15,16]. QUADAS-2 informed judgments on patient selection, biomarker measurement, diagnostic relevance, and study flow, while NOS principles informed assessment of cohort selection, comparability, and outcome reporting [15,16]. Risk of bias was summarised across four domains: selection bias, measurement bias, confounding bias, and reporting bias. Each domain was judged as low, moderate, or high risk. Low risk indicated clear recruitment, appropriate controls, reliable miRNA measurement, adequate confounder consideration, and complete reporting. Moderate risk indicated partial reporting, limited adjustment, or some methodological uncertainty. High risk indicated restricted or poorly described sampling, inadequate biomarker method reporting, insufficient control of key confounders, or incomplete or selective outcome reporting. Overall judgments were assigned by integrating domain-level ratings: low risk when most domains were low and none were high, moderate risk when one or more domains were moderate without a dominant high-risk concern, high risk when one or more key domains were high risk, and low-moderate risk when the study was generally robust but had residual concerns. The assessment was conducted independently by two reviewers, and disagreements were resolved by consensus, with third-reviewer input when required.

Result

Study Selection

**Figure 1:**
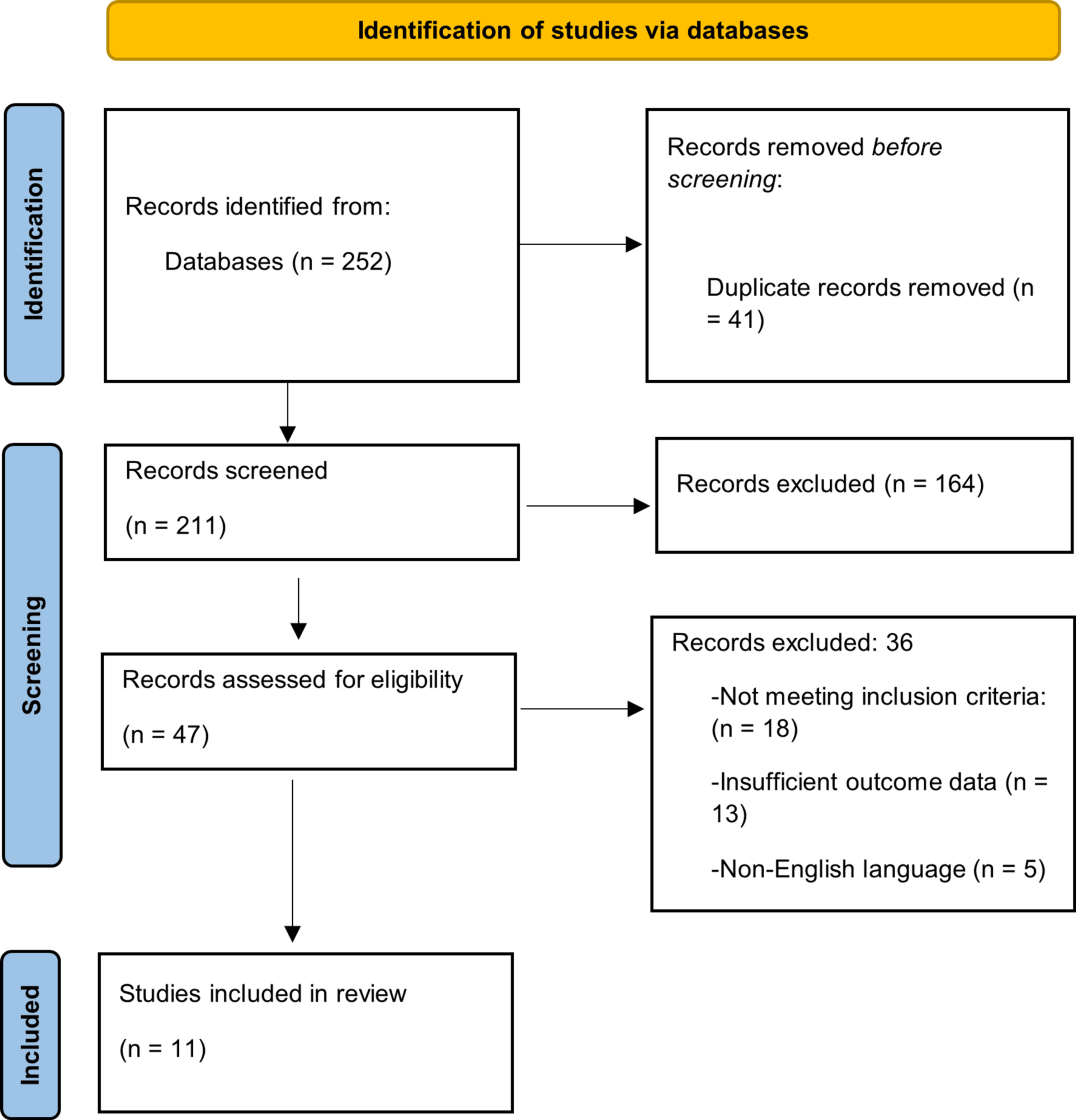
PRISMA flow chart PRISMA: Preferred Reporting Items for Systematic Reviews and Meta-Analyses.

The following databases were searched systematically for the presence of miRNA signatures in cardiovascular diseases: PubMed/MEDLINE, Scopus, Web of Science, ScienceDirect, and Google Scholar. Titles and abstracts were then screened for obvious irrelevance after the removal of duplicates. The articles were evaluated according to a set of predefined eligibility criteria: original study design; cardiovascular disease (CVD) focus; miRNA-based analysis; clearly described biological specimens; and extractable diagnostic, prognostic, predictive, severity-related, and treatment-response outcomes. Studies that failed to meet these criteria were not included. After screening, 11 studies met the eligibility criteria and were retained for the qualitative synthesis. Figure 1 (PRISMA flow diagram) describes the study selection process. 

Study Characteristics and Key Findings

The studies included in this review showed significant clinical and methodological variability, reflecting the wide range of roles of miRNAs in the mechanisms of cardiovascular diseases. A total of 11 studies explored miRNA signatures in cardiovascular risk burden, acute MI, CAD, coronary artery calcification, postoperative AF, HF, HFpEF, and PAH. Plasma was the most common biological specimen used, followed by serum, circulating blood miRNAs, and atrial tissue. Next-generation sequencing, small RNA sequencing, RT-qPCR, microarray profiling, transcriptome assays, PCR arrays, and machine-learning models were used in the studies. In general, multi-miRNA panels exhibited greater clinical discrimination than single biomarkers, especially for AMI, CAD, POAF, HFpEF, and PAH. The combined study characteristics, key findings, and validation or replication status are presented in Table 1. 

**Table 1:** Study characteristics, key findings, and validation status of included studies FHS: Framingham Heart Study, RS: Rotterdam Study, CVD: cardiovascular disease, qPCR: quantitative polymerase chain reaction, HTG: high-throughput genomics, AMI: acute myocardial infarction, STEMI: ST-elevation myocardial infarction, NSTEMI: non-ST-elevation myocardial infarction, ROC: receiver operating characteristic, LASSO: least absolute shrinkage and selection operator, CAD: coronary artery disease, PCI: percutaneous coronary intervention, GFR: glomerular filtration rate, RT-qPCR: reverse transcription quantitative polymerase chain reaction, HOMA: homeostatic model assessment, BMI: body mass index, CKD: chronic kidney disease, CT: computed tomography, CAC: coronary artery calcification, CABG: coronary artery bypass grafting, POAF: postoperative atrial fibrillation, AF: atrial fibrillation, HF: heart failure, DE miRNAs: differentially expressed microRNAs, HFpEF: heart failure with preserved ejection fraction, HFrEF: heart failure with reduced ejection fraction, GEO: Gene Expression Omnibus, LDA: linear discriminant analysis, miRs: microRNAs, PAH: pulmonary arterial hypertension, IPAH: idiopathic pulmonary arterial hypertension, PAECs: pulmonary artery endothelial cells, HUVECs: human umbilical vein endothelial cells, BMPR2: bone morphogenetic protein receptor type 2, AUC: area under the curve, RNA: ribonucleic acid, miRNA: microRNA.

Study	Study design	Population/sample size	Cardiovascular condition	Sample type	miRNA profiling/detection method	miRNAs/signature assessed	Main findings/diagnostic or prognostic value	Validation/replication status
Karlin et al., [17]	Population-based observational cohort with external replication	FHS discovery cohort: 4,440 participants; RS replication cohort: 1,999 participants	CVD risk factors, incident CVD, mortality	Plasma extracellular RNA	TaqMan advanced miRNA assays/qPCR in FHS; HTG EdgeSeq miRNA whole-transcriptome assay in RS	miR-193b-3p, miR-122-5p, miR-365a-3p, miR-194-5p, miR-192-5p, miR-193a-5p	Six-miRNA signature associated with ≥5 CVD risk factors. miR-193b-3p, miR-194-5p, and miR-193a-5p replicated in RS. Eight miRNAs were associated with all-cause mortality in FHS	Externally replicated; selected miRNAs were replicated in the Rotterdam Study after discovery in the Framingham Heart Study
Wang et al., [18]	Prospective biomarker discovery and validation study with 5-year follow-up	27 healthy controls, 64 AMI patients, 20 reperfusion patients; validation cohort: 10 AMI patients and 10 controls	Acute MI; STEMI/NSTEMI; reperfusion	Plasma	Small RNA sequencing; ROC analysis; LASSO; Cox regression; Kaplan-Meier analysis	miR-296-5p, miR-660-3p; also miR-208a-3p, miR-208b-3p, miR-499a-5p, miR-1-3p, miR-133b	Forty high-diagnostic-performance miRNAs had an AUC >0.85. A two-miRNA diagnostic model using miR-296-5p and miR-660-3p showed strong AMI discrimination. Two miRNAs were significantly correlated with all-cause mortality	Validated in an independent AMI-control cohort; prognostic associations require further external validation
Kanašniece et al., [19]	Prospective single-centre diagnostic study	108 CAD patients and 29 non-CAD controls; early-onset CAD n=80, late-onset CAD n=28, controls n=29	Early- and late-onset CAD	Plasma	Next-generation sequencing; LASSO; random forest; logistic regression; ROC analysis	miR-10b-5p, miR-29c-3p, miR-142-5p, miR-320b, miR-451a, miR-486-3p, miR-625-3p	A seven-miRNA panel distinguished late-onset CAD with very high accuracy, AUC 0.9924, and early-onset CAD with moderate accuracy, AUC 0.8235. Four miRNAs were downregulated in late-onset CAD versus controls	Exploratory/single-centre diagnostic finding; external validation required
Trusinskis et al., [20]	Prospective single-centre observational study	40 stable CAD patients undergoing elective PCI	Stable CAD; CAD severity; GFR/metabolic risk profile	Plasma	RT-qPCR using TaqMan miRNA assays	miR-126, miR-145, miR-155	miR-126 positively correlated with SYNTAX score. miR-155 correlated with triglycerides, C-peptide, HOMA index, BMI, and eGFR. miR-155 did not correlate with the SYNTAX score	Exploratory/single-centre association study; external validation required
Vijayaraghavan et al., [21]	Case-control observational microarray profiling study	200 CKD stage 3-5 patients screened by CT calcium scoring; microarray performed in 7 high-CAC CKD patients and 1 control	Coronary artery calcification in CKD	Serum	Agilent human miRNA microarray; hierarchical clustering; TargetScan-based target analysis	Upregulated: miR-21, miR-67, miR-390, miR-56, miR-250, miR-65, miR-13; downregulated: miR-235, miR-256, miR-226, miR-207, miR-255, miR-193	Thirteen miRNAs were differentially expressed in CKD patients with coronary artery calcification. No significant difference was observed in left ventricular function.	Exploratory microarray finding with a very small profiled subset; external validation required
Sathipati et al., [22]	Exploratory prospective CABG cohort with case-control POAF comparison and machine-learning analysis	15 CABG patients; 7 developed POAF, and 8 remained controls	Postoperative AF after CABG	Serum	Human Serum & Plasma miScript miRNA PCR array; machine learning; ROC analysis; DESeq2; pathway analysis	POAF signature: miR-19a-3p, miR-19b-3p, miR-184, miR-200a-3p, let-7a-5p, miR-124-3p, miR-423-5p, miR-96-5p, miR-100-5p, miR-17-5p	XGBoost showed the best predictive performance with a test AUC of 0.83; Random Forest test AUC was 0.76. Four upregulated miRNAs overlapped with the POAF signature: miR-96-5p, miR-184, miR-17-3p, and miR-200-3p	Exploratory machine-learning POAF signature from a small CABG cohort; external validation required
Harling et al., [23]	Prospective CABG cohort biomarker study	34 non-emergent on-pump CABG patients; 13 developed POAF, and 21 remained in sinus rhythm	Postoperative AF after CABG	Right atrial tissue; serum/plasma at 24 h pre-op, 48 h post-op, and 96 h post-op	Agilent miRNA microarray; TaqMan qRT-PCR; ROC analysis	miR-483-5p, miR-208a; 16 atrial miRNAs differentially expressed	miR-483-5p was overexpressed in atrial myocardium and preoperative serum of POAF patients. Preoperative serum miR-483-5p predicted POAF with an AUC of 0.78, sensitivity of 77.78%, and specificity of 77.27%	Exploratory prospective CABG biomarker study; independent validation required
Vu et al., [24]	Case-control circulating miRNA profiling study	24 HF patients and 24 age- and sex-matched non-HF patients	HF	Plasma	Next-generation sequencing; differential expression analysis; target gene and pathway prediction	DE miRNAs: let-7b-3p, miR-92b-5p, miR-145-3p, miR-206, miR-664a-5p, miR-1307-5p, miR-1908-5p, miR-3074-5p; HF-enriched miRNAs: miR-589-5p, miR-30b-5p, miR-654-3p, miR-1292-5p, miR-659-5p, miR-548d-5p, miR-7847-3p	Eight miRNAs were dysregulated between the HF and non-HF groups. Seven additional miRNAs were frequently detected in HF but not in non-HF cases. Predicted pathways involved vascular development, cell cycle, and transcriptional regulation	Exploratory circulating miRNA profiling study; independent validation required
Parvan et al., [25]	Translational preclinical study with in silico clinical validation	Female Dahl salt-sensitive rats: high-salt HFpEF model n=11, low-salt controls n=15; clinical validation using GSE53437: controls n=30, HFrEF n=41, HFpEF n=19	HFpEF; HFrEF discrimination	Rat plasma; public human miRNA datasets	qPCR-based circulating miRNA panel; GEO dataset validation; LDA; ROC analysis	rno/hsa-let-7b-5p, let-7e-5p, miR-21-5p, miR-140-3p	A four-miRNA panel was altered in HFpEF rats and significantly discriminated HFpEF from healthy controls and HFrEF patients. Gender analysis found lower let-7b-5p and miR-21-5p in females than in males	Supported by in silico clinical validation using a public human dataset; prospective external validation required
Mone et al., [26]	Prospective interventional biomarker study	30 frail older adults with HFpEF and diabetes: 10 empagliflozin, 10 metformin, 10 insulin; 10 healthy controls	HFpEF with diabetes and endothelial dysfunction	Blood/circulating miRs	RT-qPCR; endothelial miRNA panel; 2−ΔΔCt normalization	miR-126, miR-342-3p, miR-638, miR-21, miR-92	miR-126, miR-342-3p, and miR-638 were downregulated, while miR-21 and miR-92 were upregulated in HFpEF versus controls. Empagliflozin reduced miR-21 and miR-92 after 3 months; metformin and insulin did not significantly alter the endothelial miRNA profile	Exploratory interventional biomarker finding; larger independent validation required
Lago-Docampo et al., [27]	Multicenter discovery-validation diagnostic study with functional assays	Discovery: 25 IPAH patients and 10 controls; validation/prevalidation: 110 PAH patients and 110 controls	PAH	Plasma; PAECs/HUVECs for functional assays	Small RNA sequencing; qPCR validation; LASSO/logistic regression; ROC analysis; western blot; flow cytometry; tube formation assay	Diagnostic panel: let-7a-5p, miR-9-5p, miR-31-5p; functional candidate: miR-3168	The initial screen identified 29 DE miRNAs. Seven candidates were validated. The three-miRNA panel achieved an AUC of 0.862 for PAH versus controls. miR-3168 was upregulated, reduced BMPR2 protein, and impaired angiogenesis in vitro	Discovery-validation design with a larger PAH validation/prevalidation cohort and functional validation

Across the included studies, the strongest replication or validation support was observed for selected miRNA signatures evaluated in independent or validation cohorts. Karlin et al. reported external replication of miR-193b-3p, miR-194-5p, and miR-193a-5p in the Rotterdam Study after initial discovery in the Framingham Heart Study [17]. Wang et al. validated a two-miRNA AMI diagnostic model based on miR-296-5p and miR-660-3p in an independent validation cohort [18]. Parvan et al. supported a four-miRNA HFpEF panel through in silico clinical validation using a public human dataset [25]. Lago-Docampo et al. validated candidate miRNAs in a larger PAH cohort and further supported miR-3168 through functional assays [27]. By contrast, findings from smaller single-centre or exploratory studies, including studies of coronary artery calcification in CKD, POAF prediction, general HF profiling, and stable CAD severity associations, should be interpreted as hypothesis-generating until externally validated in larger prospective cohorts [20-24,26].

Risk of Bias Assessment

Sample size, recruitment strategy, validation, control of confounders, and reporting completeness were the main factors affecting the risk of bias. Exploratory single-centre studies exhibited increased bias compared with large population-based or multicentre validation studies. The most common methodological weaknesses were small sample size, limited external validation, single-centre recruitment, incomplete adjustment for clinical confounders, heterogeneity in biospecimen type and miRNA profiling platforms, variable normalisation strategies, and potential overfitting in studies using multivariable or machine-learning models. The risk of bias was particularly increased in small POAF and calcification studies because of limited sample numbers, restricted generalisability, and insufficient independent validation. External validation or replication studies and studies using standardised sequencing or qPCR workflows were given greater weight. Generally, measurement bias was lower than selection or confounding bias; however, heterogeneity in miRNA extraction, normalisation, and analytical platforms limited direct comparability across studies. Table 2 shows the integrated risk-of-bias assessment of the included studies across selection, measurement, confounding, reporting, and overall judgment domains. 

**Table 2:** Integrated risk of bias assessment The quality and risk of bias assessment was performed using an adapted domain-based framework based on QUADAS-2 (Quality Assessment of Diagnostic Accuracy Studies-2) and Newcastle-Ottawa Scale principles.

Study	Selection bias	Measurement bias	Confounding bias	Reporting bias	Overall judgment
Karlin et al., [17]	Low	Low	Moderate	Low	Low
Wang et al., [18]	Moderate	Low	Moderate	Moderate	Moderate
Kanašniece et al., [19]	Moderate	Low	Moderate	Moderate	Moderate
Trusinskis et al., [20]	Moderate	Low	Moderate	Moderate	Moderate
Vijayaraghavan et al., [21]	High	Moderate	High	Moderate	High
Sathipati et al., [22]	High	Moderate	High	Moderate	High
Harling et al., [23]	Moderate	Low	Moderate	Moderate	Moderate
Vu et al., [24]	Moderate	Low	Moderate	Moderate	Moderate
Parvan et al., [25]	Moderate	Moderate	Moderate	Moderate	Moderate
Mone et al., [26]	Moderate	Low	Moderate	Moderate	Moderate
Lago-Docampo et al., [27]	Low	Low	Moderate	Low	Low–moderate

Distribution of Cardiovascular Conditions

Evidence was available for seven cardiovascular categories, with the highest number of studies in CAD, HFpEF, and POAF. CAD evidence included early- and late-onset CAD and stable CAD severity. HFpEF studies focused on diagnostic discrimination and endothelial dysfunction. In POAF studies, the focus was on the prediction of the perioperative course in the context of CABG. There were single studies on AMI, general CVD risk factors, coronary artery calcification in CKD, HF, and PAH. This distribution suggests that the study of miRNAs is not focused on a single cardiovascular phenotype, but rather is distributed across ischemic, arrhythmic, myocardial, pulmonary vascular, and metabolic-vascular disease pathways.

Clinical Application of miRNA Signatures Across Included Studies

**Figure 2:**
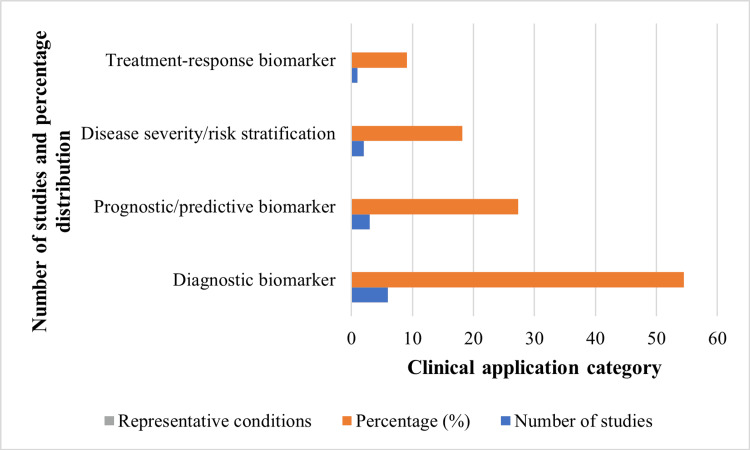
Outcome-based clinical application of miRNA signatures across included studies (n = 11)

The clinical applications of miRNA signatures varied across the included studies, with diagnostic biomarker development being the most frequent application. Diagnostic studies included AMI, CAD, HFpEF, and PAH, indicating that miRNA panels have been most actively evaluated for disease classification and case-control discrimination. Prognostic or predictive applications were less frequent and were mainly represented by mortality prediction in AMI and the prediction of postoperative AF after CABG. Disease severity and risk-stratification applications were reported in studies evaluating cardiovascular risk-factor burden, CAD severity, and coronary artery calcification, while treatment-response evidence was limited to HFpEF with diabetes. The distribution shown in Figure 2 indicates that the current evidence base is weighted toward diagnostic discovery, whereas prognostic, severity-related, and treatment-response applications remain comparatively underdeveloped and require further validation. 

Discussion

This systematic review synthesised evidence on miRNA signatures across cardiovascular phenotypes, including acute MI, CAD, postoperative AF, HF, HFpEF, PAH, coronary artery calcification, and cardiovascular risk-factor burden. The findings suggest that miRNA dysregulation is not limited to disease detection but may reflect underlying biological processes such as myocardial injury, endothelial dysfunction, vascular inflammation, oxidative stress, fibrosis, angiogenesis, calcification, and adverse remodelling. Plasma and serum were the most frequently used biospecimens, supporting the potential of circulating miRNAs as minimally invasive molecular biomarkers. A consistent pattern across the included studies was that multi-miRNA signatures generally showed stronger clinical utility than individual miRNAs, likely because cardiovascular diseases involve overlapping molecular pathways rather than single isolated mechanisms.

Several recurrent miRNAs may have cross-phenotype relevance. miR-21 was reported in coronary artery calcification and HFpEF-related endothelial dysfunction and was also included in an HFpEF discriminatory panel, suggesting possible relevance to fibrosis, inflammation, endothelial dysfunction, and cardiometabolic stress [21,25,26]. miR-126 appeared in stable CAD severity and HFpEF-related endothelial dysfunction, supporting its potential role as an endothelial and vascular injury biomarker [20]. Members of the let-7 family were reported in POAF, HF, and HFpEF contexts, suggesting potential overlap in myocardial and vascular remodelling pathways [22,25]. miR-92 was reported in HF and HFpEF with diabetes, indicating possible overlap between myocardial dysfunction and endothelial-metabolic injury [24,26]. These recurrent miRNAs may represent promising biomarker candidates, but their reproducibility remains preliminary and requires confirmation in larger, independently validated cohorts using standardised sampling, profiling, and normalisation methods.

The clinical implications differ by disease context. In acute MI, circulating miRNAs may complement troponin-based assessment by reflecting ischaemic myocardial injury and molecular stress responses, although their added diagnostic value over established biomarkers remains uncertain. In CAD, miRNA panels may support biological stratification by reflecting vascular ageing, atherosclerotic burden, endothelial dysfunction, and inflammatory remodelling. For risk stratification, extracellular plasma miRNAs associated with blood pressure, lipid traits, body mass index, glucose metabolism, and smoking-related exposure suggest that circulating miRNAs may capture cardiometabolic stress beyond conventional clinical measures [28]. In stable CAD, the association of miR-126 with angiographic disease severity and miR-155 with metabolic and renal parameters further supports a mechanistic link among endothelial dysfunction, inflammation, renal dysfunction, and atherosclerotic progression [29,30].

For postoperative AF, miRNA signatures may help identify pre-existing atrial substrate abnormalities before overt arrhythmia develops [31]. Reported associations with atrial and circulating miRNAs suggest possible links with electrical remodelling, fibrosis, inflammation, and perioperative myocardial stress. This application may be clinically useful for perioperative risk prediction after CABG, but current evidence remains limited by small cohorts and a lack of external validation [32]. In HF and HFpEF, miRNA dysregulation appears to reflect endothelial dysfunction, metabolic-vascular stress, ventricular dysfunction, and remodelling. The observed modulation of miR-21 and miR-92 after empagliflozin treatment suggests that selected miRNAs may also have value as treatment-response biomarkers, although this requires validation in larger interventional cohorts.

In PAH, the clinical and mechanistic relevance of miRNAs is supported by studies linking plasma miRNA panels with diagnosis and functional vascular biology. The association of miR-3168 with reduced BMPR2 expression and impaired angiogenic capacity supports the role of miRNAs as both biomarkers and biological mediators of pulmonary vascular remodelling [27]. The overlap between miRNA signatures and cardiovascular molecular pathways is biologically plausible because several miRNAs regulate endothelial nitric oxide signalling, oxidative stress responses, inflammatory cytokine activity, vascular smooth muscle cell proliferation and migration, angiogenesis, fibrosis, calcification, and adverse myocardial or vascular remodelling. These pathways also overlap with mechanisms described in contemporary cardiovascular molecular reviews, including hydrogen sulfide-related regulation of endothelial dysfunction, oxidative stress, vascular inflammation, angiogenesis, and cardiovascular remodelling [33]. Taken together, miRNA signatures may complement traditional clinical markers by improving biological characterisation of cardiovascular disease states, but their current role should be viewed as investigational rather than ready for routine clinical decision-making.

Limitations and Future Recommendations

The limitations of this review are mainly related to the diversity of included studies regarding cardiovascular phenotype, population size, biospecimen type, miRNA detection platform, normalisation strategy, and reported outcome measures. Formal assessment of publication bias, including funnel-plot analysis, was not performed because no meta-analysis was conducted and the included studies differed substantially in cardiovascular phenotype, study design, biospecimen source, miRNA platform, outcome definition, and reported effect measures. These differences also limited quantitative comparison of diagnostic performance metrics, such as AUCs, confidence intervals, hazard ratios, and other effect estimates across studies. Data were qualitatively synthesised, and meta-analysis was not performed because of methodological and clinical variability. Some included studies had small exploratory sample sizes, limited external validation, and inadequate adjustment for potential confounders, including age, sex, comorbidities, medication use, renal function, and disease severity. Plasma-, serum-, blood-, and tissue-based sampling may also differ biologically and technically, which can affect the comparability of reported miRNA signatures. Another important limitation is that altered miRNA expression does not necessarily indicate proportional changes at the protein-expression level. Because miRNAs act through complex post-transcriptional regulatory networks, their biomarker value may reflect disease-associated molecular regulation rather than direct clinical symptom identification. Therefore, miRNA signatures should not be interpreted as standalone tools for diagnosis or clinical symptom classification without confirmation through protein-level evidence, functional validation, conventional biomarkers, imaging, and clinical assessment.

Future research should focus on large, multi-centre, well-designed, prospective studies that use consistent approaches to miRNA extraction, normalisation, profiling, and reporting. Promising diagnostic and prognostic miRNA panels require independent validation before clinical translation. Future studies should also integrate miRNA profiles with protein biomarkers, epigenetic data, imaging findings, and clinical risk scores to improve cardiovascular risk stratification and biological interpretation. Mechanistic studies are needed to clarify how candidate miRNAs influence endothelial dysfunction, myocardial injury, fibrosis, inflammation, angiogenesis, oxidative stress, and cardiovascular remodelling.

## Conclusions

This systematic review suggests that miRNA signatures have potential as minimally invasive biomarkers across several cardiovascular diseases, including AMI, CAD, postoperative AF, HF, HFpEF, PAH, coronary artery calcification, and cardiovascular risk-factor burden. The included studies linked plasma- and serum-derived miRNAs to diagnosis, prognosis, prediction, disease severity, risk stratification, and treatment response. Multi-miRNA panels generally showed stronger clinical performance than individual miRNA markers, supporting their potential value for cardiovascular biomarker development. Several miRNAs associated with endothelial dysfunction, inflammation, myocardial injury, fibrosis, angiogenesis, vascular remodelling, and cardiometabolic stress also appear biologically relevant to cardiovascular pathophysiology. However, the current evidence remains preliminary because of substantial heterogeneity in study design, cardiovascular phenotype, biospecimen source, miRNA extraction and detection platform, normalisation approach, outcome reporting, and validation strategy. Limited external validation and lack of assay standardisation currently restrict direct clinical translation. Therefore, miRNA signatures should be considered promising investigational biomarkers rather than established clinical tools, and they require validation in large, well-designed, independently replicated cohorts using standardised analytical workflows before implementation in routine cardiovascular practice.
